# SGLT2 inhibitors and the cardiac Na^+^/H^+^ exchanger-1: the plot thickens

**DOI:** 10.1093/cvr/cvab184

**Published:** 2021-05-29

**Authors:** Yu Jin Chung, Kyung Chan Park, Sergiy Tokar, Thomas R Eykyn, William Fuller, Davor Pavlovic, Pawel Swietach, Michael J Shattock

**Affiliations:** 1 British Heart Foundation Centre of Research Excellence, King’s College London, UK; 2 Burdon Sanderson Cardiac Science Centre, Department of Anatomy, Physiology and Genetics, University of Oxford, Oxford, UK; 3 School of Biomedical Engineering and Imaging Sciences, King’s College London, UK; 4 Institute of Cardiovascular & Medical Sciences, University of Glasgow, UK; 5 Institute for Cardiovascular Sciences, University of Birmingham, UK

With the ever-mounting evidence for a profound and direct effect of SGLT2 inhibitors (SGLT2is) on the heart, understanding their mechanism of action becomes increasingly important. So, we are pleased that our paper^1^ published in this edition of *Cardiovascular Research* has generated a lively debate.^2^^,^^3^ In our work, we use a variety of methods to show that, at least in our hands, empagliflozin (EMPA) from two independent suppliers, as well as two other chemically related SGLT2is, are not potent inhibitors of the cardiac Na^+^/H^+^ exchanger-1 (NHE1) and, related to this, have no effect on intracellular Na^+^ concentration ([Na^+^]_i_) in the healthy heart. This is contrary to several previous reports (see references 1–4 in Zuurbier *et al*.^2^).

**Figure 1:**
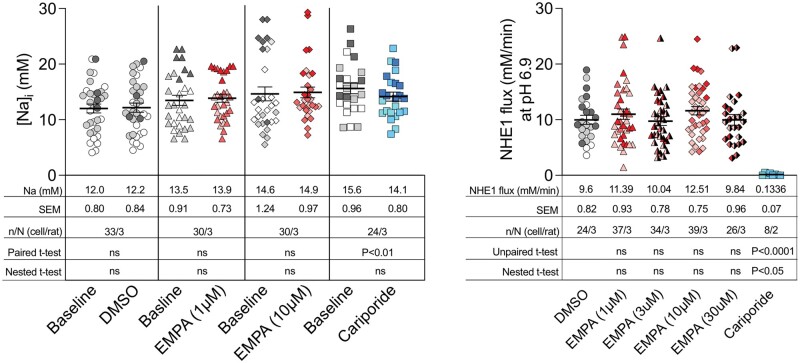
Intracellular Na measurements and NHE1 flux at pH 6.9 estimated from our original data (Chung *et al.*). Left panel: Using the calibration described by Zuurbier *et al.* the SBFI ratiometric values were converted into intracellular Na ([Na^+^]_i_). Right panel: NHE1 flux was measured at pH 6.9. In both panels, each data point represents a single observation and these are colour-coded to identify individual cell isolations. The mean values are shown in the inset tables. Using hierarchical analysis, the intraclass correlation coefficient of the [Na^+^]_i_ and NHE1 flux data were 32% and 3.2%, respectively—demonstrating the need to use hierarchical statistical test on these type of data. When tested with hierarchical (nested) *t*-tests, EMPA has no effect on [Na^+^]_i_ or NHE1 flux as previously reported. Cariporide very significantly reduced NHE1 flux whether tested by unpaired *t*-test or by hierarchical analysis. A paired *t*-test (but not nested hierarchical analysis) detects a small but significant (likely erroneous) reduction in [Na^+^]_i_ by cariporide. ns, not significant. Note: We have undertaken the retrospective Na calibration to match that of Zuurbier *et al.*, however, we recognize that this is unlikely to be reliable.

Our findings are in contrast with Zuurbier *et al.* in Amsterdam, who have responded to our work with a short letter in this issue of *Cardiovascular Research*.^2^ Their letter contains some misunderstandings and errors that warrant a response from us. However, before briefly responding to this letter, it is important to say that our labs in London and Oxford, and those of Zuurbier *et al.* in Amsterdam, have been in useful and regular correspondence over the last 6 months to try to understand the reasons for our contrasting results. We also add that we have the highest regard for the Amsterdam group, and the quality of their science, as well as a long-standing personal friendship between our groups. So, in the spirit of constructively trying to understand what underlies these apparently contrasting findings, we make the following observations:

1.* EMPA and intracellular Na concentration:* In their letter, Zuurbier *et al.* claim that, when ‘calibrated’, our SBFI data support the notion that EMPA lowers the intracellular Na concentration in isolated cells. They arrive at this conclusion by transcribing and re-analysing our SBFI ratios, read from our original figure (Figure 3A in Chung *et al.*^1^). While a *post hoc* application of an arbitrary calibration curve is unlikely to be reliable, the Amsterdam group were kind enough to share their spreadsheet and analysis with us. Unfortunately, our data have been mis-transcribed and includes some outliers that were not present in our original data set as well as other transcription errors. Nevertheless, using our real observed values and the calibration equation provided in Zuurbier *et al.*, a retrospective calibration of our data does not alter our original assertion that EMPA (1 or 10 µM) has no effect on intracellular Na^+^ (see *Figure 1*—inset table).

2. *Type I vs. Type II errors:* The Zuurbier *et al.* letter claims that they have published extensively showing evidence for SGLT2is inhibiting NHE1 activity in rabbits and mice. However, these studies, taken individually, are based on a relatively small number of observations: the primary observation of NHE1 inhibition in Baartscheer *et al.* (2017), for example, is made in five to six cells from four rabbits (Figure 2c), in Uthman *et al.* (2018) in eight cells from four mice (Figure 1), and Uthman *et al.* (2019) eight cells from five mice (Figure 5) (see references 1–3 in Zuurbier *et al.*^2^). In the recent letter by Zuurbier *et al.*, the primary observation (Figure 1A) is based on three cells from three rabbits.^2^ The [Na^+^]-lowering effect is based on similarly small sample sizes.

As David Eisner points out in a separate recent review, the use of the number of cells as the statistical sample size is valid only when comparing ‘before’ and ‘after’ drug interventions, as is the case for our cellular [Na^+^] responses (Figure 3 in Chung *et al.*^1^).^4^ The use of hierarchical statistical analysis can also limit bias due to inter-animal variability. However, the likelihood of a Type I error (i.e. false positive) increases as sample size declines, when data are clustered, or when not implementing repeated measures or hierarchical analyses.^4^

Our inferences, on the other hand, may be prone to a Type II error (false negative). We have therefore reanalysed the intracellular [Na^+^] data using hierarchical (nested) analysis (to avoid clustering bias) as well as paired *t*-tests (more likely to detect a systematic small difference). However, these analyses also fail to detect any statistically significant reduction of intracellular Na by EMPA in healthy myocytes (*Figure 1*). Our single-cell Na studies are based on 24–33 cells in each experimental group and are ‘paired’. These observations are supported by ‘unpaired’ intracellular Na measurements made using ^23^Na NMR spectroscopy in isolated rat, mouse (*n* = 6/group), and guinea pig hearts beating at physiological rates where again no changes in Na are observed. Our measurements of NHE1 activity are ‘unpaired’ as they were made in separate cells as myocytes do not usually tolerate two consecutive NH_4_ prepulses. However, hierarchical cluster analysis based on 24–39 cells from at least 3 rats (8 cells from 2 rats for cariporide) using the summary variable of NHE1 flux at pH 6.9, shows no effect of EMPA (and a significant effect of cariporide) (*Figure 1*).

3.* Specificity and sensitivity of the NHE1 assay:* The Zuurbier *et al.* letter suggests that our inability to detect an inhibition of NHE1 activity using our set-up is compromised by the ‘non-specificity’ and ‘low-sensitivity’ of our NHE1 assay. We respond to these unsubstantiated claims by arguing that the method used in Amsterdam is, in fact, more prone to be affected by non-specificity and low-sensitivity.

An NHE1-specific assay that is based on measurements of intracellular pH (pH_i_) must ensure that the only transporter responsible for producing a H^+^-equivalent flux is NHE1. We do this by eliminating any contribution from HCO_3_-dependent transporters (by buffering our solution with HEPES only). In contrast, the ‘Amsterdam’ protocol adds bicarbonate to their solutions, which inadvertently activates transporters in addition to NHE1. Thus, non-specificity is a greater concern with the Amsterdam approach. Zuurbier *et al.* point out that our recordings show a partial recovery of pH_i_ in the presence of the NHE1 inhibitor cariporide, and conclude that our system thus has a non-NHE1 component. This reasoning is, however, flawed because it ignores the fact that the dose of drug used—10 µM—is not a concentration at which cariporide is a full inhibitor. Previously, it was determined by Ch'en *et al.*^5^ that 30 µM is required to block NHE1 in rat myocytes. Nonetheless, we observe a 90% inhibition of flux in the presence of 10 µM cariporide. The pH_i_ recovery is not a sign of non-NHE1 components, but rather the product of residual NHE1 activity. In other studies, we have consistently used 30 µM cariporide to block NHE1, but in this instance, we opted for a concentration to match that of EMPA. In our NHE1 assay using HCT116 cells (Chung *et al.*, Supplement Figure 4S), we show a 97% inhibition of NHE1 flux with 30 µM cariporide.^1^
With regard to sensitivity, an NHE1 assay must ensure (i) that the transporter’s activity under control conditions is sufficiently large to detect even a small inhibitory effect of candidate drugs, (ii) that the actions of drugs are expressed in terms of flux, i.e. the most accurate functional measure of NHE1 activity, and (iii) that fluxes are compared at matching levels of transport substrate (i.e. pH_i_). With respect to the first point, we were perplexed to read that our assay was deemed to be ‘not sensitive enough’ because our NHE1 activity is too high. Our measurements peaked at 20 mM/min at low pH; as expected for rats and consistent with the literature.^6^^,^^7^ Yamamoto *et al.*^8^ have previously showed that NHE1 flux in rabbit myocytes is over four times slower than in rat myocytes. Rabbit myocytes are thus a less sensitive system to study NHE1 inhibitors. The sensitivity of NHE1 measurements in rabbits (and those in mice) by the Amsterdam protocol was further compromised by performing recordings at the unphysiological extracellular pH of 7.2–7.3, an inhibitory influence. As shown by Vaughan-Jones and Wu,^9^ the relationship between extracellular pH and NHE activity is particularly steep between pH 7.0 and 7.5, thus the use of mildly acidotic conditions will further reduce NHE activity and hence compromise its ability to resolve inhibition. At the lower NHE1 activity in rabbits, it is not surprising that even a low dose of cariporide results in an apparent block of transport; in reality, there is a small residual activity that is simply not big enough to resolve. We argue that to measure the inhibitory effect of a drug, transmembrane H^+^-equivalent flux should be calculated correctly, i.e. from the product of pH_i_ change and buffering capacity and plotted against the corresponding pH_i_ at which it was calculated to generate a pH-flux curve, as has been the standard established in our lab for over two decades. Additional transformations such as normalizations performed in the Zuurbier letter and comparing these slopes without taking into account the level of substrate (i.e. pH_i_) are problematic. NHE1 is steeply sensitive to pH_i_, therefore the effects of drugs must be compared at precisely matching levels of pH_i_.

4.* Isolated heart studies:* Both groups appear to be in agreement that EMPA has no effect on contractility in isolated hearts. The lack of a negative inotropic effect of SGLT2is has been widely reported—not only by our respective groups but also in many other studies in a wide range of models. The Uthman *et al.* and Baartcheer *et al.* studies report a fall in Na of 20–25%. Given the steep relationship between [Na^+^]_i_ and contractility (e.g. see Eisner),^10^ a reduction in [Na^+^]_i_ of this magnitude would be expected to elicit a negative inotropic response which is not observed. The lack of changes in inotropy reported in these studies is therefore surprising and suggests there are some, as yet undefined, confounding factors. Alternatively, the lack of a negative inotropic effect with this degree of [Na^+^]_i_ reduction supports our contention that intracellular [Na^+^] does not fall acutely in intact healthy hearts in response to SGLTis.

5. *SGLT2is in pathology:* While Na may not fall in healthy hearts, we agree that the beneficial effects of SGLT2is may be particularly apparent under pathological conditions. Indeed, with regard to Na fluxes, a recent study by Philippaert *et al.*^11^ has reported that EMPA blocks the slowly inactivating Na channel in failing myocytes (but not in healthy myocytes).

In this regard it is also interesting that Zuurbier *et al.* cite the excellent study of Cappetta *et al.* (see reference 5 in Zuurbier *et al*.^2^). In this study, Cappetta *et al.* report that dapagliflozin inhibits NHE in HUVECs. Studies originating from the Amsterdam group have also reported that SGLT2is can inhibit ROS production and improve NO bioavailability in HUVECs.^12^ So, while SGLT2is may inhibit NHE in endothelial cells, it is far from certain that this is a direct effect—particularly as high concentrations of NO have been shown to inhibit NHE1.^7^ Perhaps of more relevance to the present debate is Cappetta *et al.'s* observation that in cardiomyocytes, dapagliflozin had no acute effect either on systolic or diastolic Ca or on diastolic intracellular Na. They concluded that ‘*These observations suggest that the beneﬁcial effects on Ca and Na homeostasis that we observed after 6 weeks of dapagliﬂozin treatment in vivo were not caused by a direct acute modiﬁcation of [Na] and Ca ion ﬂuxes and concentrations by the drug. Therefore, in our experimental setting, dapagliﬂozin did not directly target cardiomyocyte ion transporters or channels that would otherwise determine instantaneous changes in intracellular Ca and Na*’. This therefore seems an odd paper to cite in support of their argument.

6. Conclusions: At present, we remain puzzled as to why we can find no evidence for SGLTis inhibiting NHE1 or lowering [Na^+^]_i_ in the healthy myocardium, as reported by the Amsterdam group and by Trum *et al.* (see reference 4 in Zuurbier *et al*.^2^). Zuurbier *et al.* in their recent letter have explored some differences, and we have discussed others. However, while there are clear protocol differences between our studies, we do not believe that any of them are likely to be sufficient to explain such profoundly different results. Indeed, this is the conclusion also reached by Zuurbier *et al*. The mechanisms by which SGLT2is elicit their important beneficial effects in the heart remain unresolved and therefore fertile ground for further research. We would therefore welcome suggestions from the wider community and, when we hopefully emerge from this COVID pandemic, our labs will get together in Oxford, London, and Amsterdam to try to unravel this conundrum.
